# Supplementary material for: A multifaceted interplay between virulence, drug resistance, and the phylogeographic landscape of Mycobacterium tuberculosis
Source: Microbiol Spectr. 2023 Sep 28;11(5):e01392-23. doi: 10.1128/spectrum.01392-23 (PMC10581221; doi:10.1128/spectrum.01392-23)
Supplement: Supplemental figures — Fig. S1 to S11. [file spectrum.01392-23-s0001.pdf]

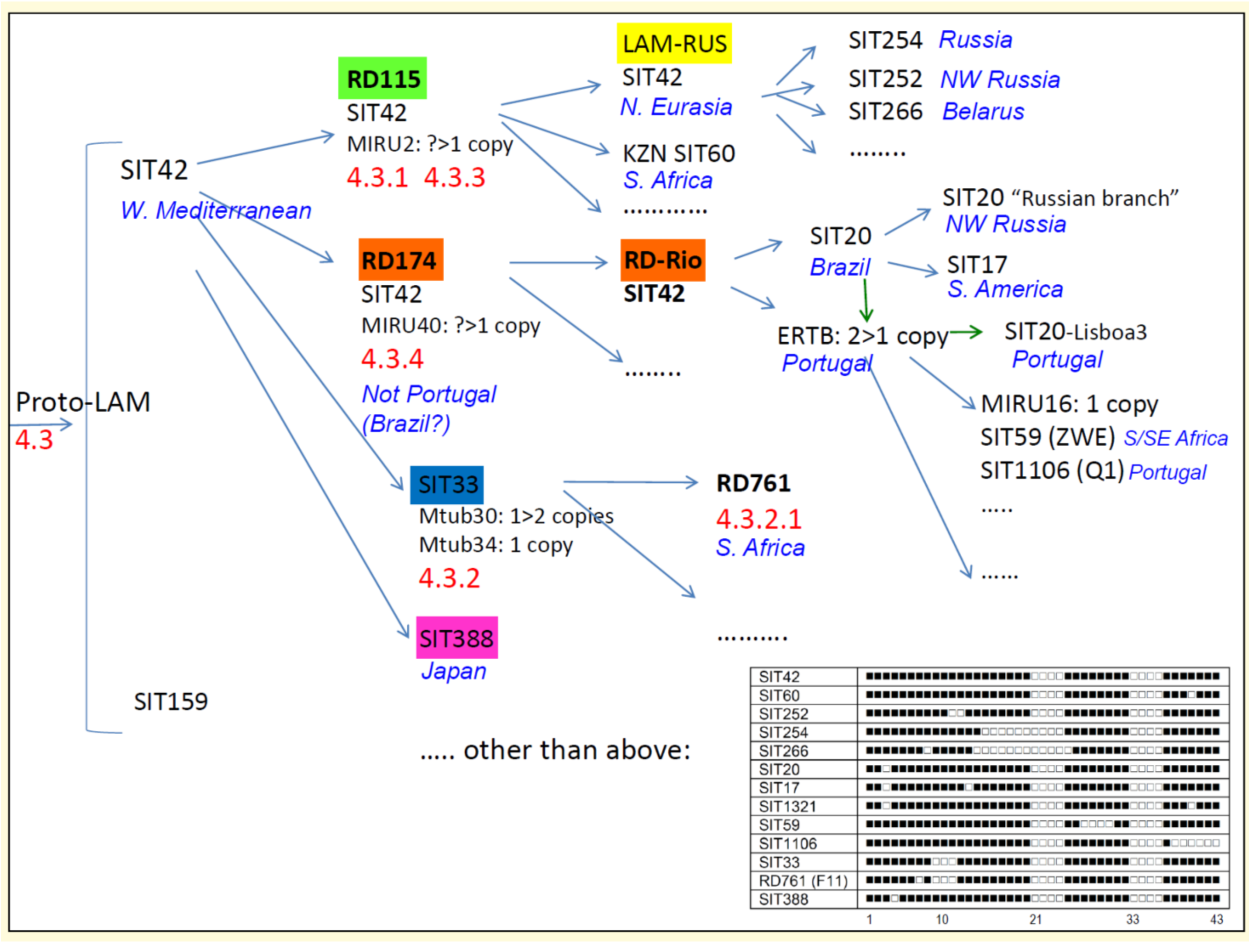

Supplementary Figure S1. Hypothesized and non-exhaustive evolutionary scenario of the LAM lineage of *M. tuberculosis*.

Barcode designations of Coll et al. (2014) are presented in red. Number of repeats in VNTR loci is shown in italic.

Reproduced from Mokrousov et al., Mol Phylogenet Evol; 2015. Copyright Elsevier NV.

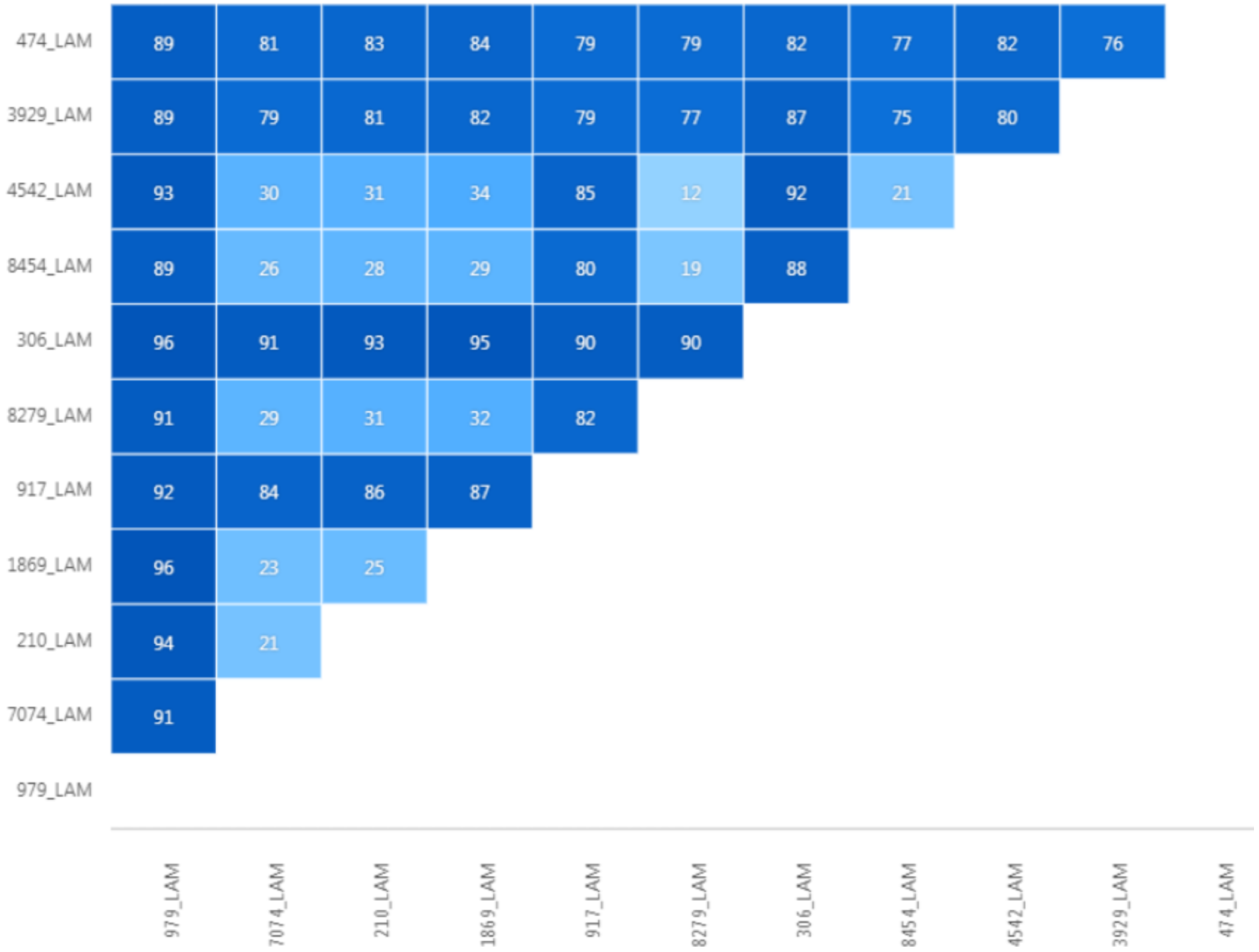

Supplementary Figure S2. Genome-wide SNP-based distance matrix for 11 LAM-RUS strains generated with SAM-TB online tool.

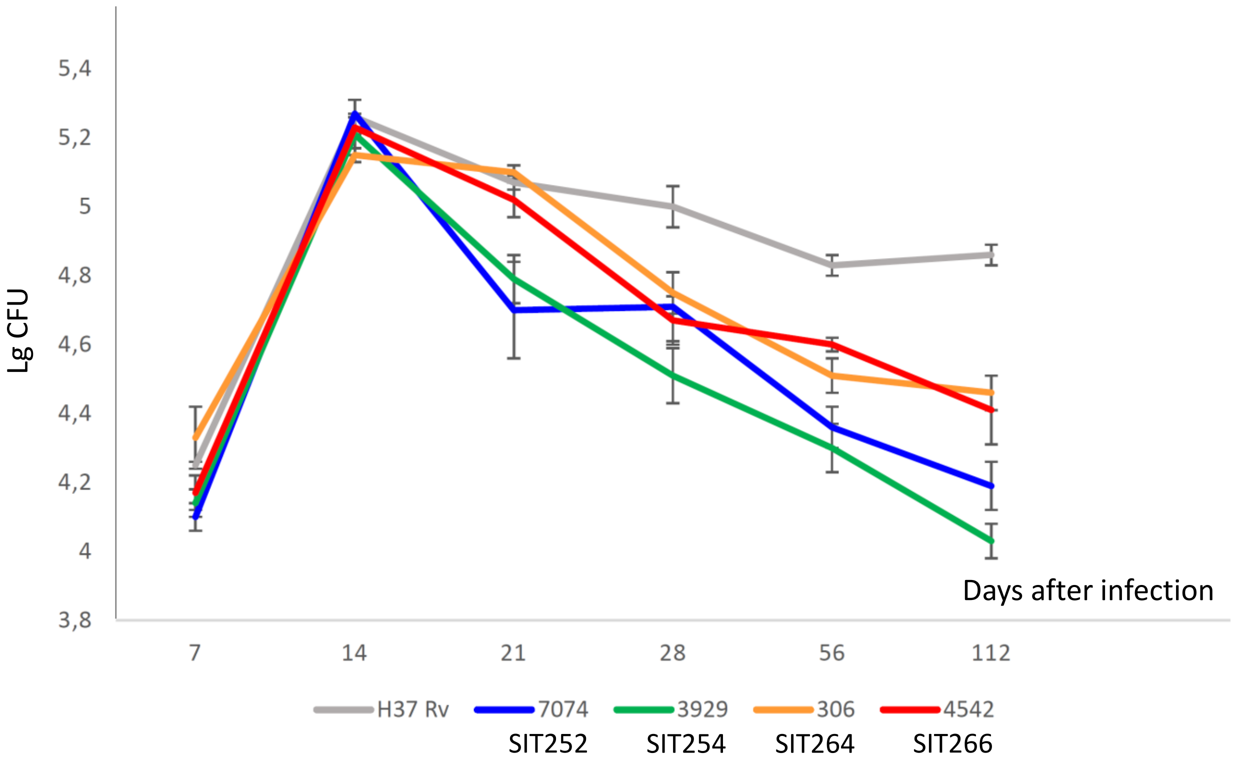

Supplementary Figure S3. Bacterial load in the spleen of mice infected with *M. tuberculosis* strains determined at different time points.

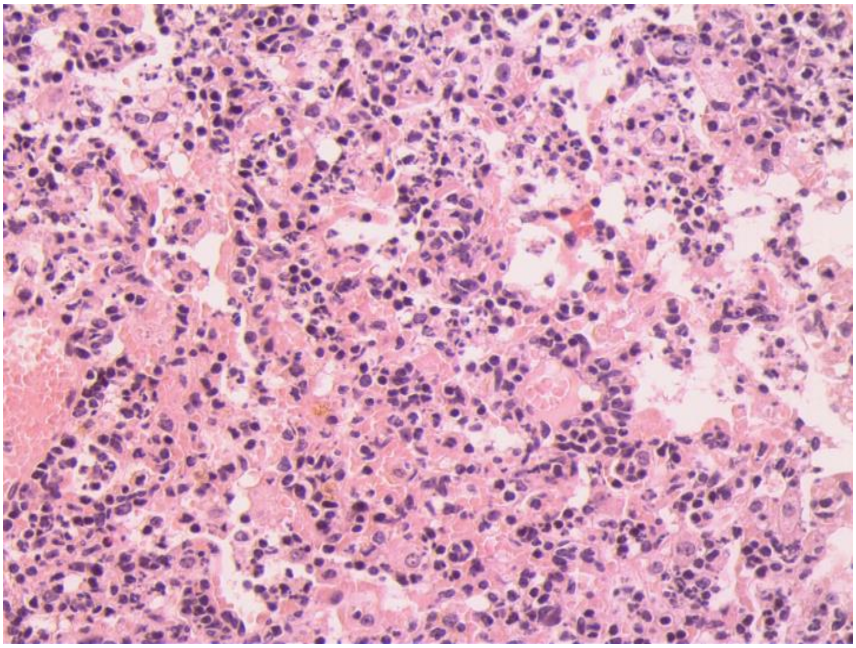

Supplementary Figure S4. Microscopic images of lung of mice on day 112 after infection with *M. tuberculosis* H37Rv. Lymphocytes, macrophages, including foamy, epithelioid cells and neutrophilic granulocytes are determined in the infiltrate. Stained with hematoxylin and eosin x600.

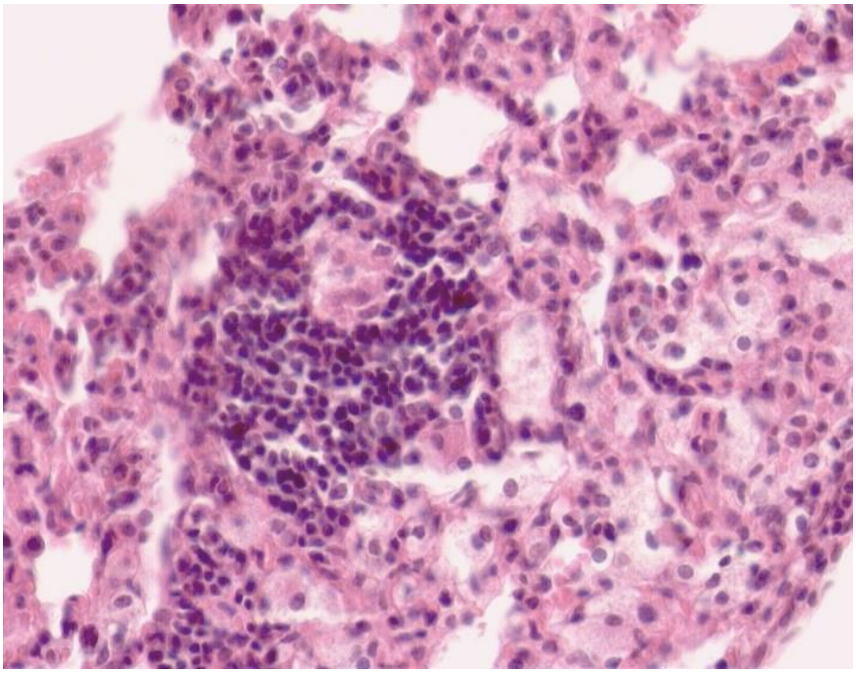

Supplementary Figure S5. Microscopic images of lung of mice on day 112 after infection with *M. tuberculosis* 306. Epithelioid cell transformation in the focus of specific infiltration. Stained with hematoxylin and eosin x600.

Supplementary Figure S6. Microscopic images of lung of mice on day 112 after infection with *M. tuberculosis* 3929. Foci of specific infiltration merging with each other without clear contours. Stained with hematoxylin and eosin x 300.

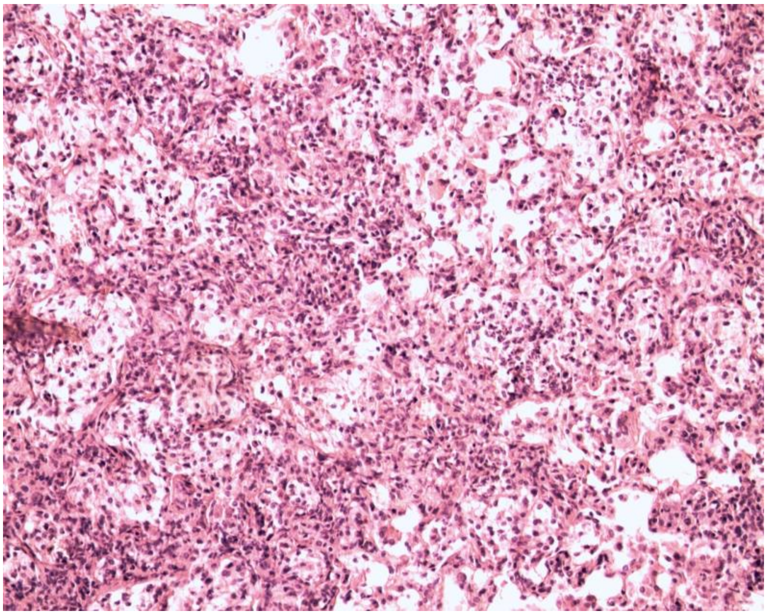

Supplementary Figure S7. Microscopic images of lung of mice on day 112 after infection with *M. tuberculosis* 4542. Serous exudate and foamy macrophages in the focus of specific infiltration. Stained with hematoxylin and eosin x 300.

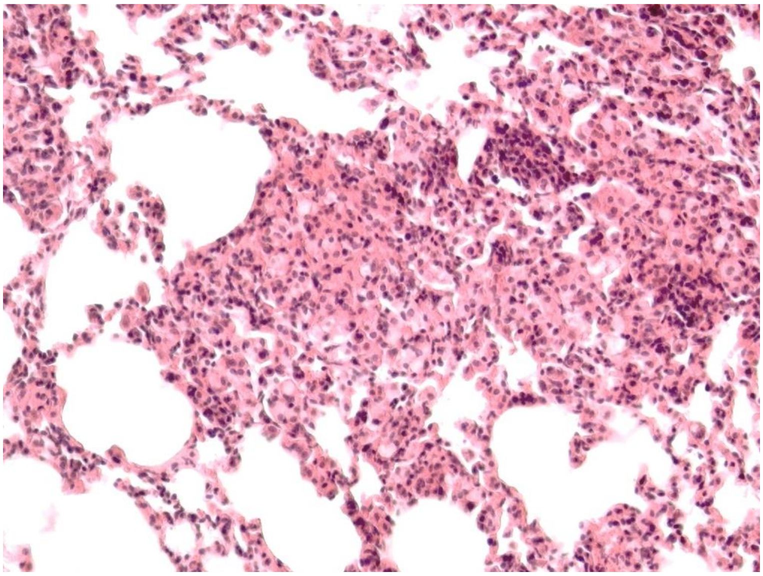

Supplementary Figure S8. Mouse lung on day 112 post infection with *M. tuberculosis* 4542. Infiltrate with protein exudate and accumulations of foamy macrophages; perivascular lymphohistiocytic infiltration. Stained with hematoxylin and eosin x 300.

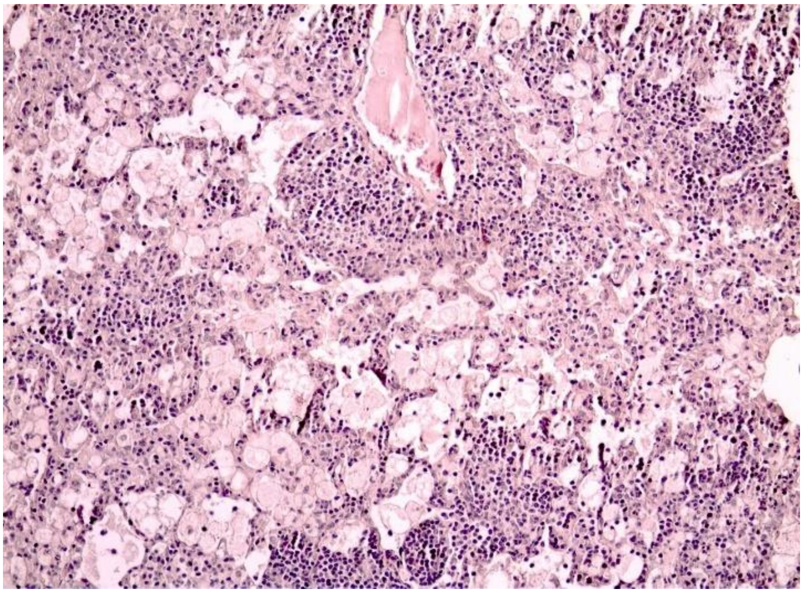

Supplementary Figure S9. Microscopic images of lung of mice on day 112 after infection with *M. tuberculosis* 3929. Perivascular lymphohistiocytic infiltration. Stained with hematoxylin and eosin x300.

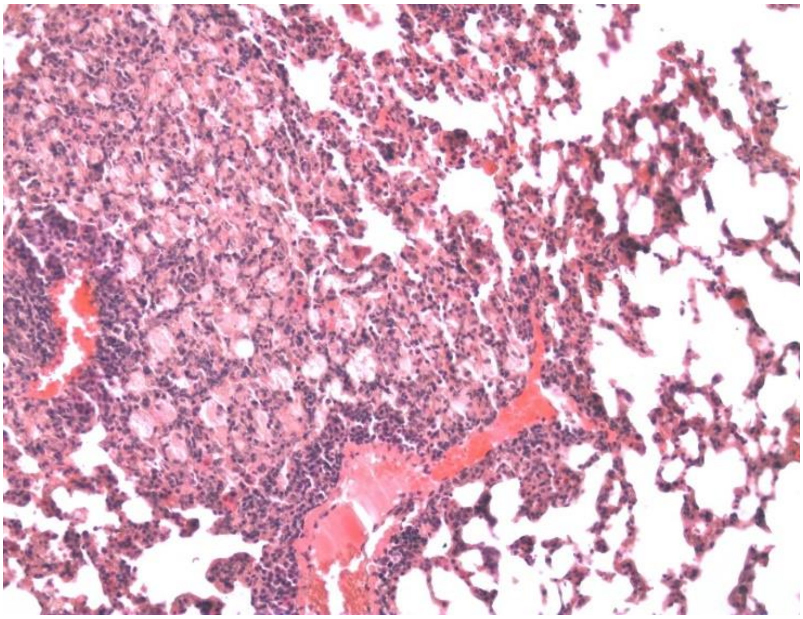

Supplementary Figure S10. Microscopic images of lung of mice on day 112 after infection with *M. tuberculosis* 4542. Small perivascular epithelioid cell granuloma in the focus of specific infiltration. Stained with hematoxylin and eosin x 600

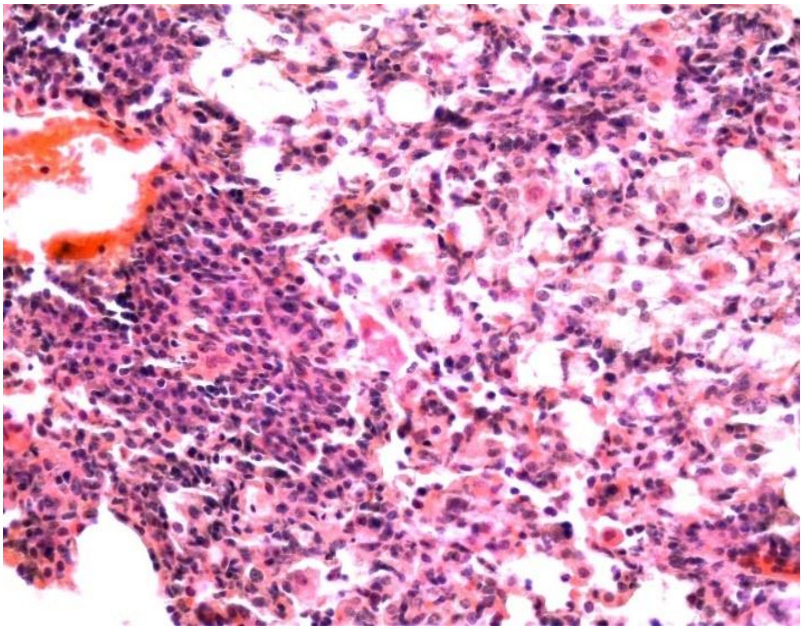

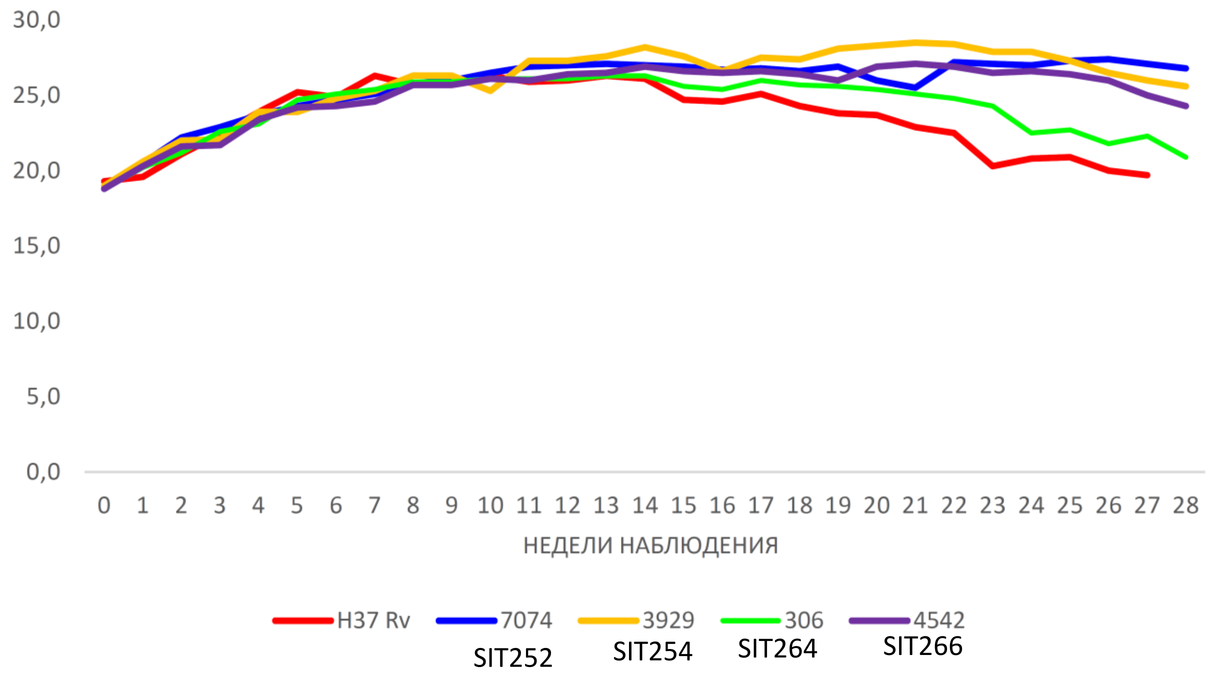

Supplementary Figure S11. Body weight changes of mice after infection with *M. tuberculosis* strains in the survival experiment.
