# Supplementary material for: A multifaceted interplay between virulence, drug resistance, and the phylogeographic landscape of Mycobacterium tuberculosis
Source: Microbiol Spectr. 2023 Sep 28;11(5):e01392-23. doi: 10.1128/spectrum.01392-23 (PMC10581221; doi:10.1128/spectrum.01392-23)
Supplement: Supplemental tables — Tables S1 to S16. [file spectrum.01392-23-s0002.pdf]

Supplementary Table S1. Assessment of the lung pathology index.

Exudative changes and score	*Productive foci and score*
aerated lungs – 0	single submiliary foci – 0.5
single airless foci – 0.25	multiple submiliary foci (≤ 20) – 1.0
Half-airless lungs – 0.5	multiple submiliary foci (> 20) –1.5
Two/third airless lungs – 0.75	single miliary foci –1.75
Airless lungs – 1.0	multiple merging submiliary and single miliary foci– 2.0
	multiple miliary foci (≤10) –2.25
	multiple merging miliary – 2.75
	appearance of small caseous necrotic foci –3.0
	extensive caseosis –4.0
	complete lung damage – 5.0

Supplementary Table S2. *M. tuberculosis* strains used in the mouse model experiment.

Mouse group / Strain	Genotypic resistance	Family and SIT	Resistance mutations	Information on strain, origin or source, and date (for clinical strain)	General information on spoligotype
1 / H37Rv	Susceptible	Lineage 4.9	-	Strain isolated in early 20th century in the USA; was used as laboratory virulent strain.	L4.9 belongs to Euro-American lineage and prevalent in US/UK early 20th century but presently is less prevalent
2 / 7074	Pre-XDR (FQ, STR, KAN, ETH, INH, RIF, PZA, EMB)	LAM SIT252	rpoB D435V, katG S315T, pncA H71R, embB M306I, inhA -12C>T, gyrA D94G, rrs514 A>C; rrs1401A>G	St. Petersburg, Russia, 2014	Высокорезистентный сполиготип генотипа LAM, эндемичен в центральной России
3 / 3929	MDR (RIF, INH, STR, KAN)	LAM SIT254	rpsL k43R, 1402C>T, rpoB D435V, katG S315T	St. Petersburg, Russia, surgery material, 2014	An endemic variant not associated with MDR, it is common in the European part of the former USSR
4 / 306	Susceptible	LAM SIT264	-	Vologda, Russia, 2018	Endemic susceptible variant, with low prevalence in the European part of the former USSR
5 / 4542	MDR (STR, INH, ETH, KAN, Cs, RIF, PZA, EMB)	LAM SIT266	rpoB H445D, katG S315T, pncA D49G, embB Q497R, embA -12C>T, rrs514A>C, rrs888G>A, inhA -12C>T, eis -12G>A, alr L113R	St. Petersburg, Russia, 2014	Highly resistant XDR spoligotype, endemic and dominant in Belarus

Supplementary Table S3. Dynamics of lung weight coefficients in C57BL/6 mice at different time-points after infection with *M. tuberculosis* strains (M±m)

Days after infection	Lung weight coefficients (conventional units)				
	M. tuberculosis strains				
	H37Rv	7074.	3929	306	4542
7<br>n=6	0.74±0.07	0.63±0.02	0.69±0.05	0.67 ±0.03	0.71±0.03
14<br>n=6	1.05±0.07	0.73±0.01<br>p_{1-2}<0,002	0.77±0.03<br>p_{1-3}<0,01	0.85 ±0.03<br>p_{1-4}<0,05<br>p_{2-4}<0,01	0.91±0.04<br>p_{2-5}<0,002<br>p_{3-5}<0,02
21<br>n=6	1.62±0,32	0.76±0.08<br>p_{1-2}<0,05	0.85±0.04<br>p_{1-3}<0,05	1.18 ±0.06<br>p_{2-4}<0,002<br>p_{3-4}<0,002	1.39±0.12<br>p_{2-5}<0,002<br>p_{3-5}<0,002
28<br>n=6	1.15±0.13	0.95±0.04	1.06±0.13	1.17±0.02<br>p_{2-4}<0,001	1.43±0.07<br>p_{2-5}<0,001<br>p_{3-5}<0,05<br>p_{4-5}<0,05
56<br>n=6	1.66±0.08	1.11±0.11<br>p_{1-2}<0,01	1.08±0.06<br>p_{1-3}<0,001	1.36±0.10<br>p_{1-4}<0,05<br>p_{3-4}<0,05	1.48±0.09<br>p_{2-5}<0,05<br>p_{3-5}<0,01
112<br>n=6	2.48±0.27	1.43±0.06<br>p_{1-2}<0,01	0,90±0.09<br>p_{1-3}<0,001<br>p_{2-3}<0,001	1.61±0.20<br>p_{1-4}<0,05<br>p_{3-4}<0,01	1.93±0.25<br>p_{3-5}<0,01

Supplementary Table S4. Significance levels (Student's t-test) for comparison between lung weight coefficients of C57BL/6 mice infected with *M. tuberculosis* strains at different time-points

Days after infectio n	*p*-values				
	M. tuberculosis strains				
	H37Rv	7074.	3929.	306	4542
7<br>n=6					
14<br>n=6	p_{7-14}<0,02	p_{7-14}<0,05		p_{7-14}<0,002	p_{7-14}<0,01
21<br>n=6	p_{7-21}<0,05		p_{7-21}<0,05	p_{7-21}<0,001<br>p_{14-21}<0,001	p_{7-21}<0,001<br>p_{14-21}<0,01
28<br>n=6	p_{7-28}<0,02	p_{7-28}<0,001<br>p_{14-28}<0,001	p_{7-28}<0,05	p_{7-28}<0,001<br>p_{14-28}<0,001	p_{7-28}<0,001<br>p_{14-28}<0,001
56<br>n=6	p_{7-56}<0,001<br>p_{14-56}<0,001<br>p_{28-56}<0,01	p_{7-56}<0,002<br>p_{14-56}<0,01<br>p_{21-56}<0,05	p_{7-56}<0,001<br>p_{14-56}<0,05<br>p_{21-56}<0,01	p_{7-56}<0,001<br>p_{14-56}<0,001	p_{7-56}<0,001<br>p_{14-56}<0,001

112<br>n=6	p_{7-112}<0,001<br>p_{14-112}<0,001<br>p_{28-112}<0,002<br>p_{56-112}<0,02	p_{7-112}<0,001<br>p_{14-112}<0,001<br>p_{21-112}<0,001<br>p_{28-112}<0,001<br>p_{56-112}<0,05		p_{7-112}<0,001<br>p_{14-112}<0,01	p_{7-112}<0,001<br>p_{14-112}<0,01

Supplementary Table S5 - Dynamics of spleen weight coefficients in C57BL/6 mice at different time-points after infection with *M. tuberculosis* strains (M±m)

Days after infecti on	Spleen weight coefficients (conventional units)				
	M. tuberculosis strains				
	H37Rv	7074.	3929.	306	4542
7<br>n=6	0.67±0.05	0.57±0.03	0,62±0.05	0.63 ±0,07	0.62±0.06
14<br>n=6	1.06±0.16	1.0±0.04	0.85±0.02<br>p_{2-3}<0,01	1.27 ±0.11<br>p_{2-4}<0,05<br>p_{3-4}<0,01	1.18±0.11<br>p_{3-5}<0,02
21<br>n=6	2.02±0.51	1.30±0.23	1.12±0.09	1.60 ±0.24	2.16±0.25<br>p_{2-5}<0,05<br>p_{3-5}<0,01
28<br>n=6	1.21±0.25	1.06±0.08	0.80±0.07<br>p_{2-3}<0,05	1.17±0.06<br>p_{3-4}<0,01	1.47±0.16<br>p_{2-5}<0,05<br>p_{3-5}<0,01
56<br>n=6	0.88±0.07	0.70±0.07	0.66±0.02<br>p_{1-3}<0,02	0.78±0.05	0.83±0.06<br>p_{3-5}<0,05
112<br>n=6	0.97±0.22	0.55±0.03	0.45±0.04<br>p_{1-3}<0,05	0.59±0.03<br>p_{3-4}<0,02	0.59±0.05

Supplementary Table S6. Significance levels (Student's t-test) for comparison between spleen weight coefficients of C57BL/6 mice infected with *M. tuberculosis* strains at different time-points.

Days after infectio n	*p*-values				
	M. tuberculosis strains				
	H37Rv	7074.	3929.	306	4542
7<br>n=6					
14<br>n=6	p_{7-14}<0,05	p_{7-14}<0,001	p_{7-14}<0,001	p_{7-14}<0,001	p_{7-14}<0,002
21<br>n=6	p_{7-21}<0,05	p_{7-21}<0,02	p_{7-21}<0,001<br>p_{14-21}<0,02	p_{7-21}<0,01	p_{7-21}<0,001<br>p_{14-21}<0,01
28<br>n=6		p_{7-28}<0,001	p_{21-28}<0,02	p_{7-28}<0,001	p_{7-28}<0,001<br>p_{21-28}<0,05

56<br>n=6	$p_{21-56}<0,05$	$p_{7-56}<0,02$<br>$p_{14-56}<0,01$<br>$p_{21-56}<0,05$<br>$p_{28-56}<0,01$	$p_{14-56}<0,001$<br>$p_{21-56}<0,001$	$p_{14-56}<0,01$<br>$p_{21-56}<0,01$<br>$p_{28-56}<0,001$	$p_{7-56}<0,05$<br>$p_{14-56}<0,02$<br>$p_{21-56}<0,001$<br>$p_{28-56}<0,01$
112<br>n=6		$p_{14-112}<0,001$<br>$p_{28-112}<0,01$	$p_{7-112}<0,05$<br>$p_{14-112}<0,001$<br>$p_{21-112}<0,001$<br>$p_{28-112}<0,002$	$p_{14-112}<0,001$<br>$p_{21-112}<0,002$<br>$p_{28-112}<0,001$<br>$p_{56-112}<0,01$	$p_{14-112}<0,001$<br>$p_{21-112}<0,001$<br>$p_{28-112}<0,001$<br>$p_{56-112}<0,02$

Supplementary Table S7 - Dynamics of lung pathology index in C57BL/6 mice at different time-points after infection with *M. tuberculosis* strains

Days after infecti on	Lung pathology index (conventional units)				
	M. tuberculosis strains				
	H37Rv	7074.	3929.	306	4542
7<br>n=6	0,96±0,04	0.5±0.13<br>$p_{1-2}<0,01$	0.71±0.04<br>$P_{1-4}<0,002$	0.71 ±0.04<br>$p_{1-4}<0,002$	0.83±0.04<br>$p_{1-5}<0,05$
14<br>n=6	2.42±0.13	1.08±0.22<br>$p_{1-2}<0,001$	1.21±0.22<br>$p_{1-3}<0,001$	1.65±0.13<br>$p_{1-4}<0,002$	1.63±0.04<br>$p_{1-5}<0,001$
21<br>n=6	2,96±0,13	2.42±0,13<br>$p_{1-2}<0,02$	2.33±0.13<br>$p_{1-3}<0,01$	2.79±0.13<br>$p_{3-4}<0,05$	2.96±0.13<br>$p_{2-5}<0,02$<br>$p_{3-5}<0,01$
28<br>n=6	3.13±0.09	3.08±0.01	3.0±0.13	2.92±0.18	3.17±0.04
56<br>n=6	3.46±0.04	3.0±0.09<br>$p_{1-2}<0,001$	2.83±0.04<br>$p_{1-3}<0,001$	2.96±0.09<br>$p_{1-4}<0,001$	2.92±0.04<br>$p_{1-5}<0,001$
112<br>n=6	3,63±0,04	3.08±0.04<br>$p_{1-2}<0,001$	3.04±0.09<br>$p_{1-3}<0,001$	3.08±0.04<br>$p_{1-4}<0,001$	3.0±0.09<br>$p_{1-5}<0,001$

Supplementary Table S8. Significance levels (Student's t-test) for comparison between lung pathology index of C57BL/6 mice infected with *M. tuberculosis* strains at different time-points.

Days after infecti on	*p*-values				
	M. tuberculosis strains				
	H37Rv	7074.	3929.	306	4542
7<br>n=6					
14<br>n=6	$p_{7-14}<0,001$	$p_{7-14}<0,01$	$p_{7-14}<0,05$	$p_{7-14}<0,001$	$p_{7-14}<0,001$
21<br>n=6	$p_{7-21}<0,001$<br>$p_{14-21}<0,02$	$p_{7-21}<0,001$<br>$p_{14-21}<0,001$	$p_{7-21}<0,001$<br>$p_{14-21}<0,01$	$p_{7-21}<0,001$<br>$p_{14-21}<0,001$	$p_{7-21}<0,001$<br>$p_{14-21}<0,001$
28<br>n=6	$p_{7-28}<0,001$<br>$p_{14-28}<0,002$	$p_{7-28}<0,001$<br>$p_{14-28}<0,001$<br>$p_{21-28}<0,001$	$p_{7-28}<0,001$<br>$p_{14-28}<0,001$<br>$p_{21-28}<0,01$	$p_{7-28}<0,001$<br>$p_{14-28}<0,001$	$p_{7-28}<0,001$<br>$p_{14-28}<0,001$

56<br>n=6	p_{7-56}<0,001<br>p_{14-56}<0,001<br>p_{21-56}<0,01<br>p_{28-56}<0,01	p_{7-56}<0,001<br>p_{14-56}<0,001<br>p_{21-56}<0,01	p_{7-56}<0,001<br>p_{14-56}<0,001<br>p_{21-56}<0,01	p_{7-56}<0,001<br>p_{14-56}<0,001	p_{7-56}<0,001<br>p_{14-56}<0,001<br>p_{28-56}<0,002
112<br>n=6	p_{7-112}<0,001<br>p_{14-112}<0,001<br>p_{21-112}<0,001<br>p_{28-112}<0,001<br>p_{56-112}<0,02	p_{7-112}<0,001<br>p_{14-112}<0,001<br>p_{21-112}<0,001<br>p_{28-112}<0,001<br>p_{56-112}<0,001	p_{7-112}<0,001<br>p_{14-112}<0,001<br>p_{21-112}<0,001	p_{7-112}<0,001<br>p_{14-112}<0,001	p_{7-112}<0,001<br>p_{14-112}<0,001

Supplementary Table S9 - Dynamics of bacterial load of the lungs (lg CFU) in C57BL/6 mice at different time-points after infection with *M. tuberculosis* strains (M±m)

Days after infection	lg числа КОЕ в посевах легких				
	M. tuberculosis strains				
	H37Rv	7074.	3929.	306	4542
7<br>n=6	4,01±0,04	3,62 ± 0,08<br>p_{1-2}<0,002	4,03 ± 0,07<br>p_{2-3}<0,01	4,11 ± 0,06<br>p_{2-4}<0,001	3,79 ± 0,13<br>p_{4-5}<0,05
14<br>n=6	5,52±0,04	5,19 ± 0,05<br>p_{1-2}<0,001	5,28 ± 0,06<br>p_{1-3}<0,01	5,38 ± 0,04<br>p_{1-4}<0,05<br>p_{2-4}<0,02	5,36 ± 0,03<br>p_{1-5}<0,01<br>p_{2-5}<0,02
21<br>n=6	5,72±0,11	5,1 ± 0,14<br>p_{1-2}<0,01	5,09 ± 0,05<br>p_{1-3}<0,001	5,58 ± 0,09<br>p_{2-4}<0,02<br>p_{3-4}<0,001	5,62 ± 0,08<br>p_{2-5}<0,01<br>p_{3-5}<0,001
28<br>n=6	5,55± 0,12	5,39 ± 0,05	5,28 ± 0,13	5,4 ± 0,08	5,74 ± 0,05<br>p_{2-5}<0,001<br>p_{3-5}<0,01<br>p_{4-5}<0,01
56<br>n=6	5,81 ± 0,07	5,18 ± 0,09<br>p_{1-2}<0,001	5,12 ± 0,05<br>p_{1-3}<0,001	5,12 ± 0,1<br>p_{1-4}<0,001	5,31 ± 0,08<br>p_{1-5}<0,002
112<br>n=6	5,99 ± 0,05	5,19 ± 0,04<br>p_{1-2}<0,001	4,72 ± 0,23<br>p_{1-3}<0,001	5,3 ± 0,15<br>p_{1-4}<0,002	5,41 ± 0,11<br>p_{1-5}<0,001<br>p_{3-5}<0,05

Supplementary Table S10. Significance levels (Student's t-test) for comparison between bacterial load of the lungs of C57BL/6 mice infected with *M. tuberculosis* strains at different time-points.

Days after infection	*p*-values				
	M. tuberculosis strains				
	H37Rv	7074.	3929.	306	4542
7<br>n=6					
14<br>n=6	p_{7-14}<0,001	p_{7-14}<0,001	p_{7-14}<0,001	p_{7-14}<0,001	p_{7-14}<0,001

21<br>n=6	$p_{7-21}<0,001$	$p_{7-21}<0,001$	$p_{7-21}<0,001$<br>$p_{14-21}<0,05$	$p_{7-21}<0,001$	$p_{7-21}<0,001$<br>$p_{14-21}<0,02$
28<br>n=6	$p_{7-28}<0,001$	$p_{7-28}<0,001$<br>$p_{14-28}<0,02$	$p_{7-28}<0,001$	$p_{7-28}<0,001$	$p_{7-28}<0,001$<br>$p_{14-28}<0,001$
56<br>n=6	$p_{7-56}<0,001$<br>$p_{14-56}<0,01$	$p_{7-56}<0,001$	$p_{7-56}<0,001$	$p_{7-56}<0,001$<br>$p_{14-56}<0,05$<br>$p_{21-56}<0,01$	$p_{7-56}<0,001$<br>$p_{21-56}<0,05$<br>$p_{28-56}<0,002$
112<br>n=6	$p_{7-112}<0,001$<br>$p_{14-112}<0,001$<br>$p_{21-112}<0,05$<br>$p_{28-112}<0,01$	$p_{7-112}<0,001$<br>$p_{28-112}<0,02$	$p_{7-112}<0,02$<br>$p_{14-112}<0,05$	$p_{7-112}<0,001$	$p_{7-112}<0,001$<br>$p_{28-112}<0,05$

Supplementary Table S11 - Dynamics of bacterial load of the spleen (lg CFU) in C57BL/6 mice at different time-points after infection with *M. tuberculosis* strains (M±m)

Days after infection	lg числа КОЕ в посевах селезенки				
	M. tuberculosis strains				
	H37Rv	7074.	3929.	306	4542
7<br>n=6	4,25± 0,01	4,1 ± 0,04<br>$p_{1-2}<0,01$	4,14 ± 0,04<br>$p_{1-3}<0,05$	4,33 ± 0,09	4,17 ± 0,05
14<br>n=6	5,26 ± 0,05	5,27 ± 0,04	5,21 ± 0,06	5,15 ± 0,02<br>$p_{2-4}<0,05$	5,23 ± 0,03
21<br>n=6	5,07 ± 0,02	4,7 ± 0,14<br>$p_{1-2}<0,05$	4,79 ± 0,07<br>$p_{1-3}<0,01$	5,10 ± 0,02<br>$p_{2-4}<0,02$<br>$p_{3-4}<0,002$	5,02 ± 0,05<br>$p_{3-5}<0,05$
28<br>n=6	5,0 ± 0,06	4,71 ± 0,1<br>$p_{1-2}<0,001$	4,51 ± 0,08<br>$p_{1-3}<0,001$	4,75 ± 0,06<br>$p_{1-4}<0,02$	4,67 ± 0,07<br>$p_{1-5}<0,01$
56<br>n=6	4,83 ±0,03	4,36 ±0,06<br>$p_{1-2}<0,001$	4,30 ± 0,07<br>$p_{1-3}<0,001$	4,51 ± 0,05<br>$p_{1-4}<0,02$<br>$p_{3-4}<0,05$	4,60 ± 0,02<br>$p_{1-5}<0,001$<br>$p_{2-5}<0,01$<br>$p_{3-5}<0,01$
112<br>n=6	4,86±0,03	4,19 ±0,07<br>$p_{1-2}<0,001$	4,03 ±0,05<br>$p_{1-3}<0,001$	4,46 ±0,05<br>$p_{1-4}<0,02$<br>$p_{2-4}<0,02$<br>$p_{3-4}<0,001$	4,41 ± 0,1<br>$p_{1-5}<0,002$<br>$p_{3-5}<0,01$

Supplementary Table S12. Significance levels (Student's t-test) for comparison between bacterial load of the spleen of C57BL/6 mice infected with *M. tuberculosis* strains at different time-points.

Days after infection	*p*-values				
	M. tuberculosis strains				
	H37Rv	7074.	3929.	306	4542
7<br>n=6					
14<br>n=6	$p_{7-14}<0,001$	$p_{7-14}<0,001$	$p_{7-14}<0,001$	$p_{7-14}<0,001$	$p_{7-14}<0,001$

21<br>n=6	$p_{7-21}<0,001$	$p_{7-21}<0,01$	$p_{7-21}<0,001$<br>$p_{14-21}<0,001$	$p_{7-21}<0,001$	$p_{7-21}<0,001$<br>$p_{14-21}<0,01$
28<br>n=6	$p_{7-28}<0,001$	$p_{7-28}<0,001$<br>$p_{14-28}<0,001$	$p_{7-28}<0,02$	$p_{7-28}<0,01$<br>$p_{14-28}<0,001$<br>$p_{21-28}<0,001$	$p_{7-28}<0,001$<br>$p_{14-28}<0,001$<br>$p_{21-28}<0,01$
56<br>n=6	$p_{7-56}<0,001$<br>$p_{14-56}<0,001$<br>$p_{21-56}<0,001$<br>$p_{28-56}<0,05$	$p_{7-56}<0,01$<br>$p_{14-56}<0,001$<br>$p_{28-56}<0,02$	$p_{14-56}<0,001$<br>$p_{21-56}<0,001$	$p_{14-56}<0,001$<br>$p_{21-56}<0,001$<br>$p_{28-56}<0,02$	$p_{7-56}<0,001$<br>$p_{14-56}<0,001$<br>$p_{21-56}<0,001$
112<br>n=6	$p_{7-112}<0,001$<br>$p_{14-112}<0,001$<br>$p_{21-112}<0,001$	$p_{14-112}<0,001$<br>$p_{21-112}<0,01$<br>$p_{28-112}<0,002$	$p_{14-112}<0,001$<br>$p_{21-112}<0,001$<br>$p_{28-112}<0,001$<br>$p_{56-112}<0,02$	$p_{14-112}<0,001$<br>$p_{21-112}<0,001$<br>$p_{28-112}<0,01$	$p_{14-112}<0,001$<br>$p_{21-112}<0,001$

Supplementary Table S13. Correlation between bacterial load of the lungs and lung pathology index estimated by Pearson correlation coefficient (r)

Strain	SIT	r	P	Comment
H37Rv		0.5326	0.07466	non significant large positive relationship
7074	SIT252	-0.1126	0.7275	non significant very small negative relationship
3929	SIT254	-0.1417	0.6605	non significant very small negative relationship
306	SIT264	0.5155	0.08631	non significant large positive relationship
4542	SIT266	0.6278	0.02885	significant large positive relationship

Supplementary Table S14. Type I and II changes in the lungs

Changes in the lungs	Relative number of mice with type 1 changes	Relative number of mice with type 1I changes
Reducing airiness by >30%	10 / 12	3 / 12
Presence of neutrophilic granulocytes in the exudate	10 / 12	3 / 12
Peribronchial and perivascular aggregations	6 / 18	18 / 18

Note. For greater clarity, fractions representing the relative numbers of mice with both types of changes are reduced to a common denominator.

Supplementary Table S15. Characteristics of the severity of TB in mice at day 196 after infection (survival experiment)

Group and number of mice	Strain	Lethality (%)	Lung weight coefficient (conventional units)	Lung pathology index (conventional units)	Cumulative average damage index
2<br>n=19	7074	5	1.77±0.10	3.20±0.06	3.32
3<br>n=19	3929	5	2.22±0.18<br>$p_{2\text{-}3}<0.05$	3.32±0.05<br>$p_{2\text{-}3}<0.01$	3. 51
4<br>n=10	306	50	3.38±0.17<br>$p_{2\text{-}4}<0.01$<br>$p_{3\text{-}4}<0.05$	3.80±0.05<br>$p_{2\text{-}4}<0.01$<br>$p_{3\text{-}4}<0.01$	19.06
5<br>n=19	4542	5	2.62±0.13<br>$p_{2\text{-}5}<0.01$<br>$p_{4\text{-}5}<0.01$	3.33±0.05<br>$p_{2\text{-}5}<0.05$<br>$p_{4\text{-}5}<0.01$	3.65

Supplementary Table S16. Genes with significant (in silico) mutations specific for 7074 SIT252 compared to 4542 SIT266

(A) mutations specific for strain 7074 (SIT252)

pos	type	gene label	aa change	SIFT *P*	function	class	Comment from Mycobrowser and otherreferences
1127119	Nonsyn SNP	Rv1008 tatD	C11R	0.00	deoxyribonuclease TatD	[4-Cell wall and processes]	DNase involved in protein export. This sec-independent pathway is termed tat for twin-arginine translocation system. This system mainly transports proteins with bound cofactors that require folding prior to export.<br>TatD DNases are conserved proteins in a variety of organisms and are considered potential virulence factors in *Plasmodium falciparum* and *Streptococcus pneumoniae* [48].<br>TatD DNases in *Trueperella pyogenes* are involved in biofilm formation and required for virulence during infections. Biofilms formed by mutants showed a significantly reduced thickness and biomass, produced a lower bacterial load in the spleen of mice and compromised virulence [48].<br>Two TatD DNases were identified in each of parasites Trypanosoma evansi and Trypanosoma brucei, and have efficient DNA hydrolysis activity. Host neutrophil extracellular traps (NETs) induced by the parasites could be hydrolyzed by native and recombinant TatD DNases. NET disruption was prevented, and the survival rate of parasites was decreased, in the presence of the DNase inhibitor aurintricarboxylic acid. These data suggest that trypanosomes can counteract host innate immune responses by active secretion of TatD DNases to degrade NETs [47].
1575044	Nonsyn SNP	Rv1399c nlhH	T256P	0.03	non lipolytic carboxylesterase NlhH	[8-Intermediary metabolism and respiration]	Latent infections impede tuberculosis eradication due to the long-term potential for reactivation. Dormant Mtb has reduced enzymatic activity, but hydrolases that remain active facilitate pathogen survival. Tallman et al. [45] targeted Mtb esterases, a diverse set of enzymes in the serine hydrolase family, and studied their activities using both activity-based probes (ABPs) and fluorogenic esterase substrates. They identified five esterases that remained active in dormant Mtb, including LipM (Rv2284), LipN (Rv2970c), CaeA (Rv2224c), Rv0183, and Rv1683. Three of these, CaeA, Rv0183, and Rv1683, were catalytically active in all three culture conditions. Fluorogenic probes additionally revealed LipH (Rv1399c), Culp1 (Rv1984c), and Rv3036c esterase activity in dormant and active cultures [45]

1884260	Nonsyn SNP	Rv1662 pks8	E853K	0.01	polyketide synthase	[2-Lipid metabolism]	Potentially involved in some intermediate steps for the synthesis of a polyketide molecule which may be involved in secondary metabolism/ Essential gene for in vitro growth of H37Rv.<br>*Pseudocercospora fijiensis* is the causal agent of the highly destructive black Sigatoka disease of banana. Previous research has focused on polyketide synthase gene clusters in the fungus, given the importance of polyketide pathways in related plant pathogenic fungi. A polyketide synthase gene cluster required for pathogenicity of *Pseudocercospora fijiensis* on banana [46]
4270285	Nonsyn SNP	Rv3807c	A18V	0.00	decaprenyl phosphoryl -5- phosphorib ose phosphatas e	[4-Cell wall and processes]	Possible conserved transmembrane protein. Essential gene for in vitro growth of H37Rv in a MtbYM rich medium, by Himar1 transposon mutagenesis. Required for survival in primary murine macrophages, by transposon site hybridization.<br>In Mycobacterium tuberculosis the decaprenyl-phospho-d-arabinofuranose (DPA) pathway is a validated target for the drugs ethambutol and benzothiazinones. To identify other potential drug targets in the pathway, we generated conditional knock-down mutants of each gene involved using the TET-PIP OFF system. dprE1, dprE2, ubiA, prsA, rv2361c, tkt and rpiB were confirmed to be essential under non-permissive conditions, whereas rv3807c was not required for survival [38]
754147	Nonsyn SNP	Rv0658c	L88R	0.01	integral membrane protein	[4-Cell wall and processes]	This region is a possible MT-complex-specific genomic island
1495139	Insert nt pos. 576 frameshi ft	Rv1328 glgP			glycogen phosphoryl ase	[8-Intermediary metabolism and respiration]	Phosphorylase is an important allosteric enzyme in carbohydrate metabolism. Enzymes from different sources differ in their regulatory mechanisms and in their natural substrates. However, all known phosphorylases share catalytic and structural properties.<br>In Korea, the Mycobacterium tuberculosis K-strain is the most prevalent clinical isolates and belongs to the Beijing family. Two proteins, Mb1363 (probable glycogen phosphorylase GlgP) and MT2656 (Haloalkane dehalogenase LinB) were most abundant after phagocytosis of M. tuberculosis K-strain by the human monocytic cell line U-937 [44]
1756485	Insert nt pos.104 1 frameshi ft	Rv1551 plsB1			acyltransfer ase PlsB	[2-Lipid metabolism]	Triacylglycerol biosynthesis is associated with Mtb dormancy. Dormant Mtb accumulates triacylglycerol (TAG) utilizing fatty acids obtained from macrophage lipid droplets. The Rv1551 (PlsB1) gene is annotated as a putative glycerol-3-phosphate acyltransferase (GPAT) in the Mtb genome. GPAT catalyzes the first step of the glycerophospholipid biosynthetic pathway that synthesizes the lipid

							precursors for triacylglycerol biosynthesis. It was observed that E. coli cell lysates expressing Rv1551 displayed increased synthesis of phosphatidylglycerol, phosphatidylethanolamine and cardiolipin from radiolabeled glycerol-3-phosphate and fatty acyl-coenzyme A precursors. When cultured in medium supplemented with long-chain fatty acids, E. coli expressing Rv1551 exhibited significantly higher viable cell counts during the exponential and stationary phases. These results suggest that Rv1551 displays function as a GPAT by enhancing the synthesis of phospholipids from exogenously provided fatty acids in E. coli cell lysates. Rv1551 is a functional GPAT that catalyzes the initial step of glycerophospholipid biosynthesis in the mycobacterial cell [39]
2497153	Intergenic-130	Rv2224c caeA			carboxylesterase A	[4-Cell wall and processes]	Disruption of this gene provides a growth advantage for in vitro growth of H37Rv, by analysis of saturated Himar1 transposon libraries. Required for growth in C57BL/6J mouse spleen, by transposon site hybridization (TraSH) in H37Rv. Required for survival in primary murine macrophages, by transposon site hybridization (TraSH) in H37Rv. Mutant is mildly attenuated in C57BL/6 mice [49]. M. tuberculosis H37Rv Rv2224c transposon mutant shows growth defect in C57BL/6 mice co-infected with mutant and wild-type; is attenuated in C57BL/6 and RAG -/- mice; is more susceptible to lysozyme; shows growth defect in C57BL/6 bone marrow macrophages and secretion of cytokines and chemokines is reduced.<br>Rv2224c is required for bacterial survival in mice. To evaluate the role of the MT2282 in pathogenesis, the gene was deleted from the M. tuberculosis genome. BALB/c mouse aerosol infections showed reduced colony-forming unit loads in lungs and spleens and less lung pathology for the DeltaMT2282 mutant. High dose intravenous infection of mice with the mutant resulted in a significantly delayed time to death compared with the wild type or complemented mutant. These results indicate that MT2282 encodes a cell wall-associated carboxylesterase, which is required for full virulence of M. tuberculosis [40].<br>Mycobacterium tuberculosis Rv2224c modulates innate immune responses. Central to the success of Mycobacterium tuberculosis (Mtb) as a pathogen is its ability to evade host immunity and to establish a chronic infection. Its primary intracellular niche is within macrophages, the underlying molecular mechanisms are poorly understood. Rv2224c, a cell envelope-associated predicted protease, is critical for Mtb virulence. Disruption of Rv2224c led to prolonged survival of infected mice and highly reduced lung pathology. Absence of Rv2224c enhanced

							host innate immune responses, compromised the intracellular survival of Mtb in macrophages, and increased its susceptibility to lysozyme. Rv2224c activity promotes processing and extracellular release of the Mtb protein, GroEL2 [43].<br>Mycobacterium tuberculosis can persist in macrophage phagosomes that acidify to a pH of approximately 4.5 after activation of the macrophage with gamma interferon. A screen of 10,100 M. tuberculosis transposon mutants for mutants hypersensitive to pH 4.5 led to the discovery of 21 genes whose disruption attenuated survival of M. tuberculosis at a low pH. Acid-sensitive M. tuberculosis mutants with transposon insertions in Rv2136c, Rv2224c, ponA2, and lysX were hypersensitive to antibiotics, sodium dodecyl sulfate, heat shock, and reactive oxygen and nitrogen intermediates, indicating that acid resistance can be associated with protection against other forms of stress. The Rv2136c, Rv2224c, and ponA2 mutants were attenuated in mice [49]<br>Dormant Mtb has reduced enzymatic activity, but hydrolases that remain active facilitate pathogen survival. Tallman et al. [45] targeted Mtb esterases, a diverse set of enzymes in the serine hydrolase family, and studied their activities in active, dormant, and reactivating cultures. They identified five esterases that remained active in dormant Mtb, including LipM (Rv2284), LipN (Rv2970c), CaeA (Rv2224c), Rv0183, and Rv1683. Three of these, CaeA, Rv0183, and Rv1683, were catalytically active in all three culture conditions. Fluorogenic probes additionally revealed LipH (Rv1399c), Culp1 (Rv1984c), and Rv3036c esterase activity in dormant and active cultures [45]

(B) mutations specific for 4542 SIT266

position	type	gene label	aa change	SIFT *P*	Function	Class	Comment from Mycobrowser and other references
1704929	Nonsyn SNP	Rv151 2 epiA	D279E	0.00	nucleotide-sugar epimerase EpiA	[8-Intermediary metabolism and respiration]	Probably involved in nucleotide-sugar metabolism. Essential gene for in vitro growth of H37Rv in a MtbYM rich medium, by Himar1 transposon mutagenesis. Non-essential gene for in vitro growth of H37Rv, by analysis of saturated Himar1 transposon libraries. Essential gene for in vitro growth of H37Rv, by Himar1 transposon mutagenesis.

204489	Nonsyn SNP	Rv0173 lprK	L142S	0.00	Mce family lipoprotein LprK	[4-Cell wall and processes]	involved in host cell invasion. mRNA also identified by microarray analysis and down-regulated after 24h and 96h of starvation. Required for growth in C57BL/6J mouse spleen, by transposon site hybridization (TraSH) in H37Rv. Required for survival in primary murine macrophages, by transposon site hybridization (TraSH) in H37Rv.
2244079	Nonsyn SNP	Rv1999c	V354M	0.00	transporter	[4-Cell wall and processes]	Possibly transporter involved in transport of undetermined substrate (possibly cationic amino acids) across the membrane: so responsible for the translocation of the substrate across the membrane.
2798950	Nonsyn SNP	Rv2488c	A644V	0.00	LuxR family transcriptional regulator	[10-Regulatory proteins]	Involved in transcriptional mechanism.<br>A particular family of genes, the mclxs (Mycobacterium cyclase/LuxR-like genes), which codify for a particular and nearly mycobacterial-exclusive combination of protein domains. mclxs genes were found to be pseudogenized by frameshift-causing insertion(s)/deletion(s) in a considerable number of M. tuberculosis complex strains and clinical isolates. Lopes-Santos et al. [50] analysed the pattern of frameshift-causing mutations in a group of M. tuberculosis isolates while taking into account their microbial-, patient- and disease-related traits. The logistic regression-based analyses have revealed disparate effects associated with the transcriptional inactivation of two mclx genes. mclx3 (Rv2488c) presents a higher tendency for pseudogenization among isolates from patients born on the Western Pacific area, and from isolates causing extra-pulmonary infections [50].<br>Expression of a subset of heat stress induced genes of mycobacterium tuberculosis is regulated by 3',5'-cyclic AMP. Mycobacterium tuberculosis (Mtb) secretes excess of a second messenger molecule, 3',5'-cyclic AMP (cAMP), which plays a critical role in the survival of Mtb in host macrophages. Although Mtb produces cAMP in abundance, its exact role in the physiology of mycobacteria is elusive. Choudhary et al. [37] analyzed the expression of 16 adenylate cyclases (ACs) and kinetics of intracellular cAMP levels in Mtb during in vitro growth under the regular culture conditions, and after exposure to different stress agents. They observed a distinct expression pattern of these ACs which is correlated with intracellular cAMP levels. Interestingly cAMP levels are significantly elevated in Mtb following heat stress, whereas other stress conditions such as oxidative, nitrosative or low pH do not affect intracellular cAMP pool in vitro. A significant increase in expression by >2-fold of five ACs namely Rv1647, Rv2212, Rv1625c, Rv2488c and Rv0386 after heat

							stress further suggested that cAMP plays an important role in controlling Mtb response to heat stress [37].<br>Transcriptome analysis revealed a small number of changes in gene expression in aerobic growth, with Rv2488c and Rv1971 being over 40-fold up-regulated. [41].
3224230	Delete frameshift (nt pos.451)	Rv2915c			hypothetical protein Rv2915c	[11-Conserved hypotheticals]	Conserved hypotheticals
3662039	Intergenic -27/-23	Rv3279c/Rv3280 birA/accD5			bifunctional biotin operon repressor/biotin-/propionyl-CoA carboxylase subunit beta	[8-Intermediary metabolism and respiration]/[2-Lipid metabolism]	Rv3280 is a key enzyme in the catabolic pathway of odd-chain fatty acids, isoleucine, threonine, methionine, and valine. mRNA identified by microarray analysis and down-regulated after 24h and 96h of starvation. Essential gene for in vitro growth of H37Rv in a MtbYM rich medium, by Himar1 transposon mutagenesis. Essential gene for in vitro growth of H37Rv, by analysis of saturated Himar1 transposon libraries. Essential gene for in vitro growth of H37Rv, by Himar1 transposon mutagenesis.<br>The Rv3281 open reading frame is co-transcribed with Rv3280 in the mycobacterial cell, and the level of epsilon-protein was highest during the log phase and decreased during the stationary phase [42].
3662040	Intergenic-28/-22	Rv3279c/Rv3280 birA/accD5			bifunctional biotin operon repressor/biotin-/propionyl-CoA carboxylase subunit beta	[8-Intermediary metabolism and respiration]/[2-Lipid metabolism]	
